# A Frank Statement on the Health Economics Franchise

**DOI:** 10.4103/0970-0218.43223

**Published:** 2008-10

**Authors:** Manicassamy B Soudarssanane

**Affiliations:** Department of Preventive and Social Medicine, JIPMER, Pondicherry - 605 006, India. E-mail: drmybase@gmail.com

India has achieved notable progress in health after its commitment to the Alma Ata declaration and especially after the revolutionary steps listed in the National Health Policy 2002(1)—particularly in fund allocations. The latest time-bound National Rural Health Mission (NRHM)(2) has ensured a great surge in taking health care closer to the underprivileged populations. Despite this appreciable progress, commitment in further areas demands urgent attention in a competitive environment.

The 35^th^ Annual National Conference of Indian Association of Preventive and Social Medicine (IAPSM), JIPMER, Pondicherry (January 2008) focused on several key issues confronting health economics in India around the theme “Health Economics: Adequate Allocation and Resource Redistribution”. The aim was to focus attention towards creating active awareness even among public health and preventive medicine specialists and thereby to other disciplines as well. Discussions concentrated on several theoretical as well as practical methods of health financing, monitoring of health fund flow, and assessing the outcome not only in terms of cost-benefit or cost-effectiveness but also in terms of the actual fraction of health finance that effectively reaches the underprivileged public.

Great success was achieved in this process of creating awareness starting with the purposeful, all embrasive, and practically oriented inaugural speech(3) given by the Union Minister of Health and Family Welfare dwelling on various issues of health financing, the social objectives of NRHM, and their achievements. Several experts in the field of health economics touched upon key issues on the theme, which was very well received by the packed audience.

The objective of this statement is two-fold; one, to reassure the theoretical and practical aspects of the plenary sessions of the conference and two, to discuss in detail the yet untouched areas in public health and preventive medicine with a stress on professional and social awareness in health economics. The erstwhile importance given to the public health services historically have receded substantively due to several factors, particularly the inventions of newer and more effective antibiotics. However, public health problems particularly among the vast sections of the interior and underprivileged population (inclusive of tribal, hilly, and desert terrains) continue to increase—more as a result of the inaccessibility of health services, despite the heavy stress on the principles of primary health care. The emergence of newer diseases further complicates this social and physical inaccessibility. The heavier public health investments should concentrate on increasing the accessibility through the establishment of primary health centers (PHC) for every 5,000 people and strengthening the availability of health services by upgrading the facilities in each PHC (including a requirement for non communicable diseases) through the availability of adequate essential drugs as well as provisions for ambulances for timely referral and follow-up. In fact, out of sheer necessity, strengthening in terms of provision of essential biochemical, microbiological, and pathological investigations as well as digital X-ray facilities and ultrasonography is a must at this population level. Accordingly, computed tomography (CT) scans and magnetic resonance imaging (MRI) facilities along with higher levels of other investigations earlier mentioned are a must at the community health center levels, which should be for every 20,000 to 30,000 people. The parallel development of specialty training is necessary. With regard to economics, this may seem huge; however, take away the undue emphasis on disproportional sub-specialty developments across the country and this is a practical proposition. The large drain of funds with miniature returns leading to huge deficits can thus be redirected ushering positive utilization of resources and definitive socio-economic returns to the community and the country.

There is a strong epidemiological basis to provide a battery of social workers, ophthalmic assistants, physiotherapists, and extension health educators along with the necessary equipment support even at the 5,000 people level. Even the currently available beds at PHC levels can only be made functional with all the above listed strengthening; otherwise, the present status of extreme non utilization will continue to lead to a virtual financial loss. Periodical revisions of the requirements of personnel, equipment, and other facilities should be built into the system. It should be possible to work out a minimum per capita health expenditure level (other than the private and out-of-pocket expenditure) that is required to balance positive cost-effectiveness.(4) The actual calculation of this level (upgradable periodically) may not be within the scope of this discussion; however, efforts are required in this direction. In place of the existing preventive services, which have averaged to only a few aspects like immunization, minimum reproductive and child health care, etc., a complete package of total services should be encouraged with parallel medical and paramedical personnel. It is well-known that the array of prevention services at the peripheral health institutions saves millions of lives as compared with the saving of a few thousand lives at the specialty and sub-specialty levels. Therefore, a bold proportional allocation is perhaps the only alternative. Quite often the policy framework is advised and directed more by a body of other specialty and sub-specialty personnel with nil or negligible representation from public health and preventive medicine fields, which leads to a biased siphoning of funds to these levels. Several administrative reforms in the health administrative hierarchy are needed to ensure a cost-effective utilization of skilled manpower. Creation of a network of Indian Health Services (IHS) on the pattern of the Indian Administrative Services could be the administrative limb of this concept. The present system of senior health administrators performing parallel clinical functions is a great drain on administrative skilled manpower. Policy decisions thereby become ad-hoc and based on inadequate discussions. The whole administrative machinery is dependent upon fewer decisions made in less time after clinical work rather than a considered, definitive administrative option. Reforms toward the option for pure administrative work at senior administrative levels would require 100% availability for administrative decision-making and care in implementation processes that would realistically reflect on the health situations of the community. Adult education at these levels would create awareness that adequately considered health administrative decisions could save thousands or even millions of lives in a single pen stroke compared with clinically saving a handful of lives one after the other. It is prudent that a theoretical situation of all sub specialists placed non functional for a long period (even a month or so) would not significantly alter the health status of the community in terms of mortality and morbidity; but if the same theoretical non functionality is applied to peripheral level public health and preventive medicine institutions, surely the effects would be very damaging.

Even with a population of 5,000, if on average the general morbidity at any given point in time is 15% (750) and if about one-fifth of these people would require consultation (150/day), there is a strong case for providing a qualified medical officer for a population of 5,000. Further, coupled with the availability of field visits and especially health education activities the requirements would be at least two medical officers for every 5,000 people. The financial requirements seem to be huge but these are manageable if there is a realistic need-based apportioning of available funds. The importance of public health and preventive medicine services should attract doctors to work in the rural underprivileged areas compared with the already explained returns in averting mortality and morbidity. Medical officers posted in PHCs/ rural areas should receive heavy compensation in terms of rural allowance (preferably non taxable) that should 2 to 3 times higher than the salary of a sub specialist in the urban area. Monetary compensation is definitely one of the efficient measures in attracting qualified personnel to underprivileged areas. Furthermore, apparently closely related areas are an investment in 1) universal primary, secondary, and higher secondary education–not only in terms of enabling awareness but also in terms of timely demand and utilization of health services and 2) food production and public distribution system. Hence, parallel apportioning of funds in the education sector and food is an essential health financing element. Likewise, apportioning of funds into basic social requirements like housing, food, and water supply have to be strengthened consciously.

The well considered directions of the Bhore Committee concentrated on the development of health services below the district level.(5) The Committee even recommended the cessation of the proliferation of hospitals until a sufficient infrastructure below the district level is developed. There has been evidence that measures concentrating on the primary prevention are much more cost-effective compared with measures related to secondary and tertiary preventions justifying the development of infrastructure necessary to strengthen primary prevention activities at all levels.(6) The overall socio-economic development would be much more effective given primary prevention measures. In fact, measures that concentrate only on secondary or tertiary prevention tend to end in a whopping negative financial balance.

The country's investment in training at the specialist level concentrates lopsided on unnecessary numbers of specialists and sub-specialists who are required only at certain referral levels. There is a huge paucity of developing specialty expertise in community medicine, obstetrics and gynecology, pediatrics, ophthalmology, ENT, psychiatry, anesthesiology, and dentistry even as per publications by the Ministry of Health and Family Welfare.(7) Unfortunately, this imbalance is only accentuated in the further sanctioning of post-graduate courses and in the number of post-graduate seats thereof. This leads to the direct negative suction of funds and decreased cost-effectiveness as the country does not produce enough specialty expertise for epidemiologically obtaining morbidity and mortality in terms of disability adjusted life years lost (and its equivalent in financial loss). Orientation towards the practice of preventive medicine should form a compulsory part of all post-graduate disciplines; short term workshops in health management and health economics should be incorporated not only at the post-graduate level but also at the junior and senior faculty levels in all disciplines in terms of adult education principles. A conscious effort to create awareness towards such an orientation has to be made; the actual practice would emerge hopefully. Recent recommendations in undergraduate training further make the situation grave. For example, the unjustifiable constriction of Community Medicine teaching for undergraduates both in terms of quantum and assessment would lead to not only decreasing awareness but also dampening of initiatives even within the profession. The unseen financial loss thereof will be colossal.

In conclusion, awareness in Health Economics should occupy a ‘must know’ category(8) in both the undergraduate and post-graduate curriculum. Operational research in the area would bring newer and more effective ways of resource utilization and socio-economic development. Evidence-based judicial allocation of resources should become a reality.
